# Inflammatory myofibroblastic tumor of hard palate: a lesion of extreme rarity

**DOI:** 10.11604/pamj.2021.38.267.28236

**Published:** 2021-03-16

**Authors:** Naman Kirit Pandya, Utsav Umang Bhatt

**Affiliations:** 1 Sharad Pawar Dental College and Hospital, Datta Meghe Institute of Medical Sciences Sawangi, Wardha, Maharastra, India,; 2 Government Dental College and Hospital, Ahmedabad, Gujarat, India

**Keywords:** Palatal perforation, inflammatory myofibroblastic tumor, aggressive, root resorption

## Image in medicine

A 19-year young female patient presented with the chief complaint of palatal perforation since 15 days. No significant medical or familial history. No h/o of trauma. On clinical examination (A) a single round perforation was present on hard palate extending antero-posteriorly from distal surface of upper left 1^st^ premolar to mesial surface of upper left 2^nd^ premolar and medio-laterally extending 1 mm medially from marginal mucosa of 1^st^ premolar to 1 cm laterally from mid palatine raphe. Clinically, the extension was approximately 5×5 mm with normal surrounding mucosa. No tooth mobility was noted. No signs of fluid discharge or nasal regurgitation was found. On palpation all inspectory findings were confirmed. No tenderness on palpation was present. Depth of the lesion was through and through from palatal to buccal cortex. On further radiological examination, cone beam computed tomography (CBCT) (B) showed a single round shape hypodense area between left maxillary premolars, approximately 1×1 cm in diameter. Aggressive destruction of buccal and lingual cortical plate with perforation in floor of maxillary sinus in left premolar region was noted (C and D). External root resorption was seen with both left premolars associated with the lesion. Differential diagnosis considering aggressive maxillary lesion was made as lateral periodontal cyst, traumatic injury and malignant tumor. Patient was planned for excisional biopsy with extraction of involved premolars under local anaesthesia. Histopathological examination revealed plump spindle cells arranged in fascicles and myofibroblastic cells with occasional mitosis. Immuno histochemistry (IHC) further confirms the morphological findings, which were inconsistent with the findings of inflammatory myofibroblastic tumor (IMT). Thus, IMT of oral cavity should be included as a differential diagnosis of any aggressive lesions or for any localised palatal perforations.

**Figure 1:**
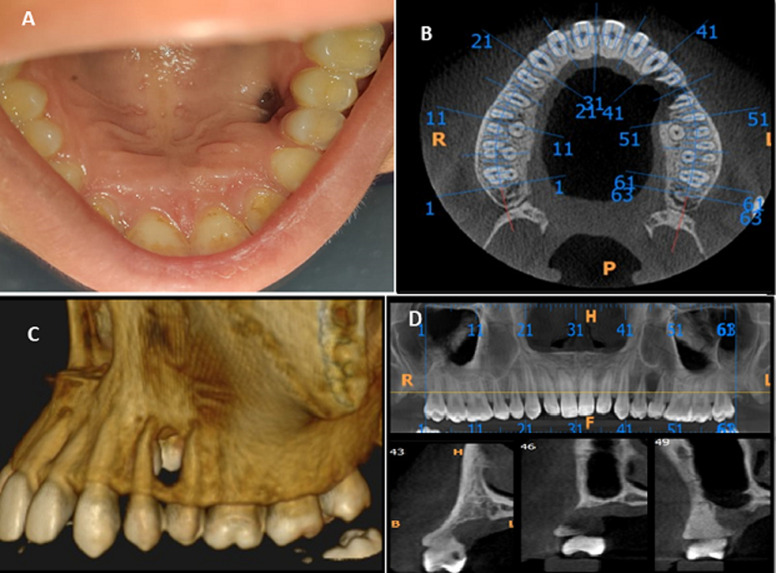
A) intraoral photograph showing palatal perforation in the maxillary left premolar region; B) axial section of cone beam computed tomography (CBCT) showing bony destruction between maxillary left premolar region; C) 3D reconstruction image showing through and through perforation in left maxillary premolar region; D) aggressive destruction of buccal and lingual cortical plate with perforation in floor of maxillary sinus in left premolar region was noted with external root resorption

